# Microbial Contamination Risks From Adjacent and Nearby Land: Evidence and Implications for Produce Safety

**DOI:** 10.1111/1750-3841.70822

**Published:** 2026-01-07

**Authors:** Tuan Le, Joseph D. Eifert, Laura K. Strawn

**Affiliations:** ^1^ Department of Food Science and Technology (0924) Virginia Tech Blacksburg Virginia USA

**Keywords:** adjacent land, cross‐contamination, human activities, produce safety, risk ranking, wild animals

## Abstract

Consumers’ demand for fresh produce is rising due to dietary preferences and public health campaigns. Fresh produce is often consumed raw or minimally processed to retain nutrient content. If contaminated, fresh produce can become a vehicle for pathogen transmission and potentially cause outbreaks. Contamination may occur within the farm border, but some evidence suggests different sources on adjacent and nearby land may be off‐farm contributors. Natural ecosystems (e.g., wildlife habitats, vegetation), agricultural activities (e.g., livestock operations, manure and compost application, wastewater discharge), residential and industrial activities (e.g., septic systems, waste discharge), and recreational uses (e.g., parks, campgrounds, golf courses) all represent potential contamination sources. Environmental pathogens from adjacent land can reach produce farms through diverse pathways influenced by meteorological drivers (e.g., rainfall, wind) or by human activities, equipment, and animal intrusion. These adjacent land inputs interact with each other and farming practices, creating complex contamination mechanisms. This paper explored microbial contamination sources from adjacent and nearby land and proposed a risk ranking model with implications for produce safety. It aimed to provide an overall understanding of the interaction between environmental and adjacent land sources as a basis for future risk assessment and management research.

## 1 Introduction

Fresh produce can become disease transmission vehicles. Microbial contamination of fresh produce is a major food safety challenge (Balali et al. 2020). Contaminated fresh produce has caused many foodborne outbreaks worldwide, burdening not only public health but also the food industry with heavy economic loss (Hussain and Gooneratne 2017). The Centers for Disease Control and Prevention (CDC) documented 972 reported fresh produce outbreaks that caused 34,674 diseases, 2,315 hospitalizations, and 72 mortalities in the U.S. from 1998 to 2013 (Bennett et al. 2018). Among these outbreaks, the most notable microbial agents were *Salmonella enterica* (21%), *Escherichia coli* O157:H7 (10%), *Cyclospora* (2%), *Campylobacter* (2%), and *Listeria monocytogenes* (1%) (Bennett et al. 2018). Yang and Scharff (2024) examined produce outbreak data from 1998 to 2020 to find that *Salmonella spp., E. coli* O157:H7, and *Campylobacter* were still the highest outbreak attributions. These authors also estimated that up to 9.2% of known pathogen‐caused foodborne illnesses were attributed to leafy greens; leafy greens were tied to 2,307,558 estimated illnesses and $5.28 billion in costs annually, and lettuces were linked to over 75.7% of leafy green foodborne illnesses and 70% of costs. In Europe, fresh produce accounts for about 10% of all foodborne outbreaks between 2007 and 2011, leading to 35% hospitalizations and 46% mortalities (Iwu and Okoh 2019). Still, the rising consumption of fresh produce creates pressure on growers and producers to minimize hazard contamination. The Food Safety Modernization Act (FSMA), under the Food and Drug Administration, established the Produce Safety Rule (PSR) in 2015 as an official effort to “minimize the risk of serious adverse health consequences or death from consumption of contaminated produce” (FDA 2015).

Fresh produce farms can be contaminated on and off their operational boundaries. Adjacent or nearby land is one of the off‐farm sources that contribute to fresh produce contamination. The FDA distinguishes “adjacent” land as land that shares a common physical border with the farm, whereas “nearby” land does not share such a physical border but is close to the farm perimeter (FDA 2024). “Adjacent” and “nearby” land will be regarded as one in this review due to their similar potential to impact produce safety. Environmental pathogens from adjacent land can be transmitted onto produce farms under the influence of single or combined meteorological sources (e.g., humidity, rain, wind) and other activities (e.g., humans, animals, equipment) (FDA 2024). Several recent fresh produce outbreaks have traced conditions and practices on adjacent land as a contributing factor: *Salmonella Newport* in red onions in 2020 (FDA 2021a), *Salmonella Enteritidis* linked to peaches in 2020 (FDA 2021b), six Shiga‐Toxin Producing *E. coli* (STEC) outbreaks associated with leafy greens (including four outbreaks between 2018 and 2020) (FDA 2019, 2021c), and a *Salmonella Typhimurium* outbreak involving cantaloupe grown during 2022 (FDA 2023a). Thus, a comprehensive understanding of the interaction between environmental and off‐farm sources from adjacent land is needed to better manage food safety risks. Some previous works provided comprehensive reviews on pathogen transmission routes with preharvest fresh produce, such as the spread of antimicrobial resistance (AR) (Iwu and Okoh 2019), or how different environmental pathways transmit pathogens between animal operations and produce crops (Leaman et al. 2022). This review aims to extend previous works by reiterating the roles of environmental, animal, and human drivers on fresh produce cross‐contamination, with a focus on adjacent land. This review also proposes a possible risk‐ranking model to prioritize contamination sources for future mitigation strategies.

## 2 Sources on Adjacent Land That Facilitate Pathogen Transfer to Produce Farms

Pathogens can spread from adjacent land to a produce farm in many ways. Understanding how pathogens travel and the conditions or drivers that influence the likelihood, intensity, or persistence of pathogens enables effective contamination control and prevention on produce farms. Under the context of contamination from adjacent land, pathogens can spread through the following (but not limited to) pathways. Table 1 provides a summary of some selected pathogen transmission sources.

**TABLE 1:** Transmission sources for major produce‐related foodborne pathogens. This table summarizes documented environmental, animal, and human‐associated sources of key pathogens (*Salmonella, E. coli* O157:H7, *L. monocytogenes, C. cayetanensis, Campylobacter)* linked to produce contamination. Sources include water, soil, wildlife, livestock, and humans, with corresponding citations indicating where each source has been reported.

			Potential sources		
Pathogens	Water	Soil	Humans and their feces	Animals and their feces	References
					
*Salmonella*				Rodents	Meerburg and Kijlstra 2007
				Beef cattle, poultry, and swine production environment	Rodriguez et al. 2006
				Reptiles, poultry, cattle, swine, rodents, birds, cats, dogs	Hoelzer et al. 2011
				Deer, turtle, and birds	Gruszynski et al. 2014
				Cattle, poultry, swine, hedgehogs	Kagambega et al. 2013
				Deer	Strawn et al. 2013a
				Turkey	Oni et al. 2015
				Feral pigs, waterfowl, deer, raccoons	Topalcengiz et al. 2020a
			Food workers		Todd et al. 2007
			—		Hedican et al. 2009
			Human waste		Im et al. 2016
			Human waste		Khanam et al. 2021
*E. coli* O157:H7				Bovine	Fukushima et al. 1999
				Various	Duffy 2003
				Beef cattle	Hussein and Bollinger 2005
				Various	Belanger et al. 2011
				Deer	Asakura et al. 2013
	Cattle and human wastewater			Various	Ahmed et al. 2015
				Cattle	Shridhar et al. 2017
				Cattle, feral pigs, waterfowl, deer, raccoons	Topalcengiz et al. 2020b
				Deer	Szczerba‐Turek et al. 2023
*L. monocytogenes*		Soil and decaying vegetation			Welshimer and Donker‐Voet 1971
		Agricultural soil			Dowe et al. 1997
				Mammals and birds	Kalorey et al. 2006
			Human waste	Various	Lyautey et al. 2007
	Agricultural water	Agricultural soil		—	Strawn et al. 2013a, Strawn et al. 2013b
		Soil and decaying vegetation			Muller‐Herbst et al. 2014
		Agricultural soil			Soni et al. 2014
				Deer and boars	Weindl et al. 2016
				Cattle	Hurtado et al. 2017
	—	Agricultural soil			Zhu et al. 2017
	Wastewater	Wastewater‐irrigated soil			Gholipour et al. 2020
				Deer and boars	Palacios‐Gorba et al. 2021
			Human waste	Various	Schoder et al. 2022
*C. cayetanensis*	Various		Infected humans	Shellfish	Ortega and Sanchez 2010
	—		—	Nonhuman primates	Ortega and Robertson 2017
	—		—		Hadjilouka and Tsaltas 2020
	Agricultural water				Durigan et al. 2020; Kahler et al. 2021; Totton et al. 2021; Naganathan et al. 2022; Reyes et al. 2023
	Agricultural and wastewater				Kahler et al. 2024
*Campylobacter*	Agricultural water				Ceuppens et al. 2015; Guevremont et al. 2017; Ssemanda et al. 2018; Mulder et al. 2020
				Birds	Hald et al. 2016
				Livestock	Salaheen et al. 2016
	—			Poultry	Kougblenou et al. 2019
				Livestock and poultry	Pires et al. 2019
	—	Pasture soil		Birds, rodents, cows	Rapp et al. 2020
		—		Poultry	Xu et al. 2021
	—			—	Reichelt et al. 2022
				Reptiles, mammals, and birds	Olvera‐Ramirez et al. 2023

*Note*: Filled cells only reflect which pathway(s) were found in the referenced literature.

Empty cells do not reflect that there is no pathway detected.

(‐) denotes similar pathway(s) from the above cell.

### 2.1 Environmental Sources

#### 2.1.1 Land Conditions

Land conditions include untouched, nature‐modified, and human‐modified characteristics of the surrounding landscape. Land conditions are challenging to control, as they are a combination of soil heterogeneity, temporal‐spatial factors (temperature, water inputs, and pathogen sources), and meteorological factors (rainfall, wind, and humidity) (Bradford et al. 2013; Strawn et al. 2013a). Pathogen transmission from adjacent land can be highly complex with soil/water runoff, soil erosion, and particle aerosolization (Bradford et al. 2013). Surface runoffs caused by rainfall are more significant when land slopes are present, especially if a produce farm is located at a lower elevation than its adjacent land. Natural or cultivated vegetation on adjacent land can affect runoff in areas surrounding farms. Grassland with better ground cover can reduce runoff depth compared to forestland with poor ground cover (Chen et al. 2018). However, forestland with higher cover than grassland might act as a natural barrier to airborne pathogen transmission. A combination of grassland and forestland is suggested for best overall coverage, as vegetation barriers can reduce the transmission of *Salmonella* and *E. coli* from animal operations to fresh produce (Glaize et al. 2021). However, vegetation might provide habitat and attract wild animals on adjacent land. Fragmentation of adjacent land vegetation might be an appropriate practice to hinder the movement of wildlife and increase the extinction of bacterial subpopulations (Liao et al. 2021a). If vegetation fragmentation is too extreme, soil surfaces may become dry due to prolonged exposure to the sun, allowing soil pathogens to become airborne with dust particles. Proximity to other land uses can create a contamination chain. Proximity to adjacent pastures and to impervious surfaces is a factor influencing the likelihood of detecting *L. monocytogenes*‐positive samples on fruit and vegetable farms, as adjacent pastures and impervious surfaces (e.g., roadside ditches) provide nesting habitats (Strawn et al. 2013a). Land use history (e.g., deforestation, agricultural development/irrigation, urbanization) is related to disease transmission (Gottdenker et al. 2014). Different land use can alter pathogen transmission mechanisms through changes in vector, host, and pathogen niche, interaction, and spatial distribution transmission (Gottdenker et al. 2014).

#### 2.1.2 Rainfall

Excessive rainfall might create overland flow that moves pathogens onto farms. Compared with other contamination sources (e.g., livestock operations, manure runoff, or wastewater effluent), rainfall might act as a lower‐risk, secondary mechanism that mobilizes pathogens already present on adjacent land. Overland flow can contaminate everything on its flow path, including produce, soil, equipment (e.g., irrigation pipelines, sprinkler systems, tools), and vehicles. The effect of rainfall and rainfall intensity on pathogen transfer and survival on produce has been extensively studied. Strawn et al. (2013a) detected a higher level of *L. monocytogenes* and *Salmonella* on adjacent land next to produce farms when water bodies, rainfall, soil erosion, and cooler weather are present. Heavy rainfall and storm events can facilitate pathogen transport in water, along with sediment that provides habitat for pathogen survival. Heavy rainfall with a high flow rate may transport pathogens up to 32 km (Strawn et al. 2013a). Raindrops may aerosolize and transfer pathogens that survive on soil or dry surfaces onto produce through splash events, allowing pathogens to be ejected when small bubbles (formed when raindrops hit the soil) burst (Joung et al. 2017). *Salmonella* was detected in splash water at heights of up to 80 cm from the soil surface and can survive for a prolonged time if splashed onto the produce surface (Lee et al. 2019). In addition to the produce surface, pathogens can internalize into produce cells when coming into direct contact with rainwater (Cevallos‐Cevallos et al. 2012). Recontamination can occur when flooded areas retain pathogens and reintroduce them into other areas when water moves and evaporates. Callahan et al. (2016) detected *E. coli* in soils up to 9 m downslope from a flooded zone within 1–3 days, and *E. coli* persistence in flooded soil was recorded up to 63 days, with persistence affected by field slope, moisture, and temperature. Increasing precipitation correlates with increased salmonellosis (Morgado et al. 2021; Manchal et al. 2024). The odds of isolating *L. monocytogenes* from soil samples at different New York produce farms increased for each millimeter increase in rainfall (Weller et al. 2015). Spinach was 3.5‐fold more likely to be contaminated with *E. coli* for every millimeter increase in rainfall in Texas and Colorado farms (Park et al. 2014). Reduced rainfall might decrease pathogen prevalence to an undetectable level (Gorski et al. 2011). *C. cayetanensis* transmission usually happens in warm and heavy rainfall months but can also occur during dry months (Almeria et al. 2019; NACMCF 2023). Donnison et al. (2011) emphasized that rainfall, combined with effluent discharged from dairy farms, increases the concentration of *E. coli* and *Campylobacter* in surface water.

#### 2.1.3 Soil

Contaminated soil can serve as both reservoirs and vehicles for pathogen transmission from adjacent land to produce farms. Soil is primarily contaminated with pathogens through surface runoff from rainfall and fecal matter from wildlife, livestock, or working animals that move across adjacent land. However, soil can also accumulate pathogens through less visible or indirect routes. Agricultural soils amended with untreated or inadequately treated manure or irrigated with contaminated water can harbor human pathogens (Zhu et al. 2017; Gholipour et al. 2020). Wastewater discharge, compost piles, and livestock housing areas on adjacent land may leach microorganisms into the soil, especially under wet conditions or where drainage flows toward crop production areas. Soil and decaying vegetation on adjacent land drainage areas or unmanaged buffer zones can provide nutrient‐rich microenvironments that promote pathogen survival and persistence (Muller‐Herbst et al. 2014). Under these conditions, pathogens may persist for extended periods, especially when protected by organic matter or soil particles that shield them from desiccation or UV exposure. Once present in soil, pathogens can move onto produce farms in several ways. Soil particles can migrate through splash events during rainfall, dust drift under dry conditions, or physical transfer via machinery, footwear, and animal intrusion. Soil composition (soil type and nutrients) can impact the abundance, diversity, and composition of bacterial communities in soil (Liao et al. 2021a). Certain micronutrients and minerals may influence pathogen persistence or competitiveness. For example, *Listeria* prevalence was associated with molybdenum levels (Liao et al. 2021b; Cook et al. 2023). Soil texture can also influence transport, as coarser soils facilitate infiltration and movement through pore spaces, while finer soils hold moisture and organic matter that enable longer microbial survival. High soil humidity supports *E. coli* survival and growth (Pang et al. 2018), and repeated wet–dry cycles can resuspend pathogens into surface runoff or dust, reintroducing microorganisms into the production environment.

#### 2.1.4 Wind

Wind facilitates pathogen transmission and deposition variably depending on the carrier (dust, vapor, water droplets, aerosols). Stagnant airflow can maintain aerosols, whereas moving airflow can transmit them (Le et al. 2024). In drier months, wind transports pathogen‐embedded dust off the ground (Fernstrom and Goldblatt 2013; Zhao et al. 2014). Iwabuchi et al. (2010) found that 48 out of 203 layer farms were positive for *Salmonella* in airborne dust, particularly in farms with high dust circulation. Schmidt et al. (2012) detected *E. coli* O157:H7 and *S. enterica* in 291 air samples (16% and 16.5%, respectively) from a cattle operation, suggesting that hide removal disperses liquid droplets containing pathogens. *Listeria* is frequently found in drains, processing environments, and portable equipment (John et al. 2020). Notably, airborne *Listeria* transmission is a concern in cattle production (Okraszewska‐Lasica et al. 2014; Dogan et al. 2023). Wind direction and elevation significantly influence the transfer of pathogens from adjacent land. Airflow moving from higher to lower elevations can carry pathogens downslope, and downwind directions transport more bioaerosols than upwind (Kumar et al. 2024). Sanz et al. (2015) reported that wind direction positively influenced *E. coli* quantity at multiple elevations around a dairy farm. When animals are not concentrated, bacteria remain more ground‐stable, and wind has a reduced transmission effect (Leaman et al. 2022). Higher wind speed can reduce *E. coli* O157:H7 due to desiccation (Benjamin et al. 2015), but strong gusts can aerosolize dust, mobilizing pathogens from contaminated soil or manure. Open poultry farms exposed to higher wind speeds and surrounding agricultural areas had an increased prevalence of Campylobacter (26.0%, 250/962) (Smith et al. 2023), suggesting that wind can cross‐contaminate between livestock and neighboring areas. Short‐term high wind speed increased *Listeria* prevalence in a mixed farm; that higher average wind speed 2 days before sampling and elevated precipitation in the preceding 25 days were statistically significant predictors of *Listeria* detection (Pang et al. 2017).

#### 2.1.5 Wild Animals

Wild animals shed pathogens through feces, contaminate water bodies, and produce fields with their movement and intrusion. Deer, birds, rodents, and other feral species can carry *E. coli* O157:H7, *Salmonella*, and *Campylobacter* (Beutin et al. 1993; Jay et al. 2007; Jay‐Russell 2013). In the 2006 spinach outbreak caused by *E. coli* O157:H7, the outbreak strain was initially isolated from cattle feces found approximately 1 mile away from the spinach field, where numerous free‐roaming feral swine were observed (Jay et al. 2007). Feral swine feces were found in crop fields and adjacent vineyards, along with evidence of intrusion, including tracks and rooting, suggesting a possible correlation between swine presence and outbreak strains (Jay et al. 2007). The same outbreak of the *E. coli* O157:H7 strain was found in over 60 avian species residing in proximity to produce fields, along with *Salmonella* and non‐O157 Shiga toxin‐producing *E. coli* (Navarro‐Gonzalez et al. 2020). Birds can also carry pathogens over long distances when migrating; for example, the 2005 *Campylobacter jejuni* outbreaks with raw peas were due to crane feces contamination (Gardner et al. 2011). However, pathogen transfer in birds might be more noticeable in big migratory birds compared to small resident birds, as 305 sampled small birds in different locations in Yuma County, AZ (one of the largest produce‐growing regions in the U.S.), did not test positive for *E. coli* O157:H7 or *Salmonella* (Fonseca et al. 2020). These studies suggest that waste deposited outside a field can reach produce without direct animal entry. Many pathogens use insects as vectors for cross‐contamination. Flies and cockroaches contaminate fruits’ and vegetables’ surfaces with *E. coli*, *Salmonella*, and *Campylobacter* (Alegbeleye et al. 2018). Additionally, flies feeding on leafy greens can internalize *E. coli* O157:H7 through mechanical breakdown of leafy green surfaces (Talley et al. 2009).

### 2.2 Human and Human Activity Sources

#### 2.2.1 Agricultural Sources

##### 2.2.1.1 Domesticated Animals

Like wildlife, domesticated animals distribute pathogens through fecal excretion, animal body contamination (fur, feathers, hooves, and hides), and movement into produce fields. Aerosolized contamination from nearby livestock operations was implicated in leafy‐green *E. coli* O157:H7 outbreaks in 2019 (FDA 2019), *Salmonella* outbreaks in 2020 linked to sheep grazing (FDA 2021a), and *Salmonella* outbreaks in 2021 linked to fugitive dust (FDA 2021b). Cattle movement creates dust (Zhao et al. 2014), causes soil compaction and increases runoff (Centeri 2022), and may spread waterborne pathogens when crossing ponds or streams (Risebro et al. 2007). Working animals that roam between animal areas and human spaces might indirectly transmit human pathogens. Dogs have been suggested to be involved in *E. coli* and *Salmonella* transmission when living in environments with livestock or livestock waste (Caldow and Graham 1998; Mateus et al. 2008; Hogg et al. 2009). Cats can carry *Salmonella* as they prey on small zoonotic species (e.g., bats, rodents, and birds) (Taylor and Philbey 2010; Salinas‐Ramos et al. 2021). Animal feeding operations (AFOs) and concentrated AFOs (CAFOs) generate large quantities of bioaerosols due to feces accumulation (Dungan 2012). Larger herd sizes increased the odds of detecting *E. coli* O157:H7 near produce regions (Benjamin et al. 2015). CALGMA (2024) specifies distancing metrics for CAFOs: 30 ft (no composting) or 400 ft (with composting) for AFOs and 1200 ft for CAFOs. Berry et al. (2015) reported that 400 ft (120 m) may be insufficient due to aerosolization and dust‐mediated transfer. *E. coli* populations were highest in pistachio orchards located 35 m (115 ft) from a poultry operation, with transmission influenced by upwind direction (Theofel et al. 2020). Livestock housing can serve as a major pathogen reservoir as fecal matter is distributed within and outside housing environments. *E. coli* O157:H7 transmission occurred via houseflies through hides, equipment, feed, and water (McGee et al. 2004; Alam and Zurek 2004; Ahmad et al. 2007). Livestock transmitted *E. coli* O157:H7 to horses in indoor conditions (Tyrnenopoulou et al. 2024). *L. monocytogenes* and *Campylobacter* were detected in feces and feed in loose‐housing and tie‐stalled dairy systems (Idland et al. 2022) and in environmental samples from housing pens and milking equipment (Latorre et al. 2009). *Salmonella* was detected in the air of poultry houses (Fallschissel et al. 2009) and in indoor and outdoor cattle/swine housing (Arnold et al. 2015). These studies demonstrate that domesticated animals serve as both primary contamination sources and amplifiers of airborne, waterborne, and mechanical transmission pathways from adjacent land to produce fields.

##### 2.2.1.2 Human Workers

Pathogens can travel with human movement or human activity, not only through direct person‐to‐person spread but also through the ways humans interact with the environment. One route is the improper excretion or disposal of human waste, which can introduce pathogens into soil, water, or irrigation systems that come into contact with crops. Another route is that farmworkers and food handlers act as vectors if sanitation practices are not followed (Beuchat and Ryu 1997; Todd et al. 2007). Temporary workers, visitors, or vehicles contaminated with pathogens may unintentionally carry them between farms or adjacent lands. This was evident during the 2006 *E. coli* O157:H7 spinach outbreak, in which harvesting equipment resembling a lawn mower picked up fecal deposits in the field, allowing a single contaminated machine to spread pathogens across a large volume of product during processing (Jay et al. 2007). Poor hygiene practices, contaminated footwear, and lack of sanitation increase these risks. When toilet or handwashing facilities are absent, insufficient, or poorly maintained, workers may be more likely to relieve themselves in nearby fields or irrigation areas, creating direct routes of pathogen introduction. Cross‐contamination also occurs when individuals travel between livestock areas, composting sites, or waste zones and produce fields without changing clothing, gloves, or footwear. Vehicles and machinery that operate on both types of land can track manure, soil, or contaminated water into crop areas. Waste collection trucks, transport vehicles, and equipment used for land preparation are examples of vectors that may carry pathogens from adjacent land into farming areas if cleaning and disinfection procedures are not followed. Vehicle movement aerosolizes road dust and soil dust, suspending and resuspending pathogens (Alex et al. 2023). EFSA BIOHAZ 2022 reported that motor vehicle transport can spread antimicrobial‐resistant bacteria and genes between farms. In operations where temporary or seasonal labor is common, the frequency of worker movement and shared equipment increases opportunities for transfer.

##### 2.2.1.3 Other Agricultural Sources

Livestock operations, manure management, composting, soil amendments, and wastewater discharge can all transfer pathogens into water bodies (used for irrigation), soil, and produce farms, especially when manure or organic waste is improperly treated. Inadequate manure treatment allows pathogens to persist in compost. Enteric pathogens persist for extended periods in slurry manure because its high moisture, low solids, and alkaline pH create a favorable environment (Manyi‐Loh et al. 2016). On the other hand, solid manure is often stored in piles within animal housing to reduce bacterial load, but this practice may introduce contamination within the animal housing. Maintaining the appropriate temperature, moisture, and pH levels of compost can be challenging, even after it is incorporated into the soil for an extended period. In 2461 composted soil samples used to amend produce, 318 were positive for at least one pathogen over 30–120 days post‐application (Ramos et al. 2021). Pathogens can leach from amendments into underlying groundwater that is later pumped into irrigation networks (Amin et al. 2013), and these risks increase when irrigation sources draw from surface waters receiving runoff or wastewater from neighboring lands (Chevez et al. 2023). Overhead irrigation systems amplify this pathway because water is sprayed directly onto edible plant tissue, allowing pathogens present in irrigation water to contact crops without additional environmental barriers (Steel and Odumeru 2004; Bhavsar et al. 2023; Wilson et al. 2024). Even when irrigation infrastructure is properly installed, runoff from adjacent land during rainfall or flood events can introduce pathogens into irrigation canals and ditches, where water is then recirculated back onto produce fields, creating a contamination feedback loop. Leaky pipes, damaged conveyance lines, or open channels further increase opportunities for pathogen entry and transport.

#### 2.2.2 Non‐Agricultural Activities

##### 2.2.2.1 Residential Activities

Residential activities can generate waste through toilet facilities, sewage or septic systems, or wastewater treatment (FDA 2024). Some produce farms, especially small or rural farms, are co‐located with residences relying on private wells and onsite septic systems. Septic systems and leach fields can serve as reservoirs for microbiological hazards that contaminate nearby irrigation water sources (Bremer and Harter 2012; Mbae et al. 2024). Onsite systems are particularly prone to affecting older or shallow wells positioned in close proximity; contamination has been reported at distances of roughly ∼30 m (Gyimah et al. 2024). As urban development spreads, farms that were once isolated may now border residential neighborhoods. CALGMA (2024) recommends 30 ft (9 m) of agricultural separation from homes or other buildings with a septic leach field in urban settings. While there is not a universal standard for an appropriate distance between residential homes and farms, and separation from agriculture and residential areas is based on local zoning laws and state regulations, a minimum distancing of 9 m (30 ft) or greater (30 m) is advised as best practice according to current knowledge (CALGMA 2024; Gyimah et al. 2024). In reality, mixed agricultural‐residential land use is common in peri‐urban regions; for example, “agrihood” communities physically integrate homes and production fields, which may introduce food safety risks if septic systems are not properly maintained (Sangroniz et al. 2024). Increased population density near farmland also increases septic system density, which has been positively associated with fecal contamination of surface and groundwater (Sowah et al. 2014; Digaletos et al. 2023). Irrigation water sourced from contaminated surface water or treated wastewater is frequently implicated in produce‐associated outbreaks (Uyttendaele et al. 2015; Allende and Monaghan 2015). Untreated residential wastewater sampled near a produce‐growing region along a river contained detectable *E. coli* (1.1 ± 5.5 CFU/100 mL), which was later transferred to soil and produce (Ali et al. 2023). Human wastewater can also contribute antimicrobial‐resistant bacteria into agricultural environments (Samreen et al. 2021). Treated municipal effluent may still contain high microbial loads; for example, Korzeniewska et al. (2013) found more than 2.7 × 10^3^ CFU/mL *E. coli* in treated municipal wastewater. Anastasi et al. (2012) found 264 *E. coli* isolates in a 20 km radius of sewage plants, of which 140 were pathogenic. Other major pathogens were also identified: Krzyzanowski et al. (2014) found that 38.9% of sewage sludge samples contained Salmonella, while Gholipour et al. (2020) found that 50% contained *Listeria*. Septic systems’ design and maintenance failure, and their proximity to surface water might have contributed to the 2013 *C. cayetanensis* pre‐packaged salad mix outbreak through groundwater intrusion from septic system leach fields, as large protozoan parasites such as *Cyclospora* are more likely to attach to soil particles during the filtration process (Kahler et al. 2024).

##### 2.2.2.2 Recreational Activities

In the U.S., recreational lands can be spatially adjacent to agricultural lands, such as those used for produce farming. Through programs such as the USDA Voluntary Public Access and Habitat Incentive Program (VPA‐HIP), owners and operators of privately held farm, ranch, and forest land can voluntarily make their land available for access by the public for wildlife‐dependent recreation (e.g., camping, boating, swimming, hunting, fishing, RV sites, golf courses, and parks) (7 CFR 1455.1 2025). These recreational activities introduce human presence, waste generation, equipment movement, and animal attraction directly into landscapes bordering produce production. When recreational areas lack adequate sanitation infrastructure, concentrated use and onsite system failures can lead to improper waste disposal and elevated bacterial loads in streams and soils. *E. coli*, *Listeria*, and protozoa were found in national park water sources and pit toilets, demonstrating that recreational traffic can create microbial reservoirs on adjacent land (Derlet and Carlson 2004; Scott et al. 2022). Forrester et al. (2017) found higher *E. coli* concentrations downstream of backpacker sites and pack stock crossings compared to upstream, with the highest downstream increase in *E. coli* of 32 CFU/mL during storm events. Similarly, Phiri et al. (2021) found *E. coli* and *Campylobacter* in drinking water and animals near 15 campgrounds. Humans can also shed pathogens into water during swimming and bathing activities. A person might discharge 0.14 g of fecal waste within 15 min of swimming, with one gram of fecal matter containing 2–9 log of Enterobacteriaceae (Gerba 2000). A person might also shed more than 5 log *Enterococci* and more than 6 log *Staphylococcus aureus* within the same 15‐min period (Elmir et al. 2007). Once introduced into water, pathogens can move beyond the point of deposition through other water activities. Mechanical disturbance of water surfaces by motorized vehicles (e.g., boats, jet skis) can facilitate the airborne transmission of pathogens by aerosolizing them into water droplets. Beyond waterborne transmission, recreational activities also create additional contamination routes relevant to produce safety. Vehicles, bicycles, fishing gear, and camping equipment can move between contaminated recreation areas and agricultural fields, potentially transferring pathogens on tires, footwear, or gear surfaces. Hunting and fishing activities leave carcass remnants and food waste that attract wildlife, increasing the likelihood of animal intrusion into produce farms. Pet access, dog walking, and off‐leash recreation can introduce fecal material from companion animals, which are known carriers of zoonotic pathogens.

##### 2.2.2.3 Industrial Activities

Industrial operations near farmland facilitate pathogen transfer mainly through wastewater discharge and runoff. The dumping of treated sewage sludge can result in the airborne transmission of pathogens within a 10 km radius, contaminating water and food sources within that radius (Dowd et al. 2000; Reilly 2001). Waste from broiler operations can spread antibiotic resistance genes to nearby cropland. Of 83 *E. coli* isolates quantified from streams draining concentrated poultry operations, 33% of the isolates were resistant to at least one antibiotic, 24% were resistant to ampicillin, 13% were resistant to cefazolin, and 8% were multidrug resistant (Amato et al. 2020), spreading antibiotic resistance genes through runoff. Industrial effluents often contain heavy metals and non‐biodegradable particles that interfere with disinfection processes by shielding pathogens from UV light or increase their interaction with disinfectants (Chahal et al. 2016). When these effluents come into contact with soil, their chemical components can alter the soil pH and disrupt native microbial communities, reducing the competitive exclusion normally provided by resident microbiota. These disturbances can create conditions that allow invading pathogens to establish and persist. *L. monocytogenes* grows optimally at pH ∼7.0 but was demonstrated to survive in acidic, nutrient‐poor sandy soil (pH 5.46) when soil microbial communities were disturbed (Spor et al. 2020). Effluents also supply organic matter that supports the formation of biofilms on surfaces such as irrigation pipes, storage tanks, and transport equipment (Ren et al. 2024). Biofilms can be protective barriers to shield pathogens from disinfectants and environmental stresses. Constructed wetlands are sometimes installed between industrial land and produce farms as a secondary wastewater treatment step; however, root internalization of *Salmonella* has been observed in wetland vegetation (Alufasi et al. 2022). Contaminated wetlands may act as a secondary source if runoff or floodwaters exit the system and enter irrigation canals or fields.

## 3 Proposal for a Risk Ranking Model for Contamination Sources on Adjacent Land That Facilitate Pathogen Transfer to Produce Farms

Through the FSMA PSR (FDA 2015), the FDA has consistently sought to enhance its efforts in assessing and controlling hazards associated with fresh produce operations. Specifically, with the 2018–2020 STEC outbreaks in the California Central Coast growing region, the FDA created the *Leafy Greens Action Plan*, with one of its specific goals being to increase awareness around adjacent land use (FDA 2023b). Until now, there has been no systematic risk assessment effort specifically targeting adjacent land uses as sources of fresh produce contamination. We propose a risk ranking model as the first step in assessing the risks associated with adjacent land uses. Risk ranking in food safety is “systematic analysis and ordering of foodborne hazards and/or foods in terms of public health risks, based on the likelihood and severity of adverse impacts on human health in a target population” (UN FAO 2020). If contamination sources from adjacent land are ranked and prioritized, then targeted mitigation strategies can be employed to reduce contamination to produce farms. The FDA recognized risk ranking as an appropriate approach in achieving its goals within and outside fresh produce commodities, with examples such as the *Produce Risk Ranking Tool* (P3ARRT) (Anderson et al. 2011) and the *Risk‐Ranking Model for Food Tracing* (RRM‐FT) (Chen et al. 2022). Risk ranking can be carried out using different approaches; however, in this section, we propose a risk ranking model for contamination sources on adjacent land using an RRM‐FT approach (Chen et al. 2022).

Our proposed Risk Ranking Model for Adjacent Land (RRM‐AL) consists of 3 sequential objectives, with the aim of systematically ranking pathogens and contamination sources from adjacent land to produce farms and applying the ranking to real‐life situations. The first objective would be to identify relevant adjacent‐land sources and key pathogen contaminants. A representative set of 11 specific adjacent land contamination sources and 5 significant pathogens (FDA 2024) can be studied as source‐pathogen pairs (Table 2). The goal of this objective is to develop an initial ranking of 5 selected foodborne pathogens under 11 sources of contamination from adjacent land to 1) identify which pathogen is most strongly driven by adjacent land sources overall, and 2) identify high‐priority source‐pathogen interactions that might or might not require further study. For example, the *presence of domesticated animals, animal housing, animal waste, and related practices* is a key source for *Salmonella*, whereas, for *C. cayetanensis, equipment and transport vehicle handling and traffic patterns* might not be a key source. A risk score can be assigned to a pathogen‐source pair based on either quantitative data (scientific literature, reports, and surveillance/outbreak data) or qualitative data (expert opinion survey). Risk scores could be weighted to put more emphasis on sources that contribute more to contamination from adjacent land.

**TABLE 2:** Ranking of eleven sources on how they contribute to the spread of five pathogens. This table presents a comparative scoring of eleven contamination sources, including domesticated animal operations, wildlife presence, waste sites, recreational and residential activities, sanitation infrastructure, agricultural water systems, worker practices, equipment traffic patterns, land features, and weather events, based on their relative ability to transmit five major produce‐related pathogens (*Salmonella, E. coli* O157:H7, *L. monocytogenes, Campylobacter, and C. cayetanensis*).

Sources Pathogens	Presence of domesticated animals, animal housing, animal waste, and related practices	Presence of wild animals or presence of animal attractants or habitats	Presence of waste or trash storage areas	Presence or evidence of recreational and residential activities	Toilet facilities, sewage or septic systems, or wastewater treatment facilities	Agriculture water sources or systems, and related practices	Worker practices (including presence of untreated or improperly treated human waste)	Equipment and transport vehicle handling and traffic patterns	Land features (e.g., topography, vegetation) and land use	Weather events: rainfall	Weather events: wind	Final score
*Salmonella*												
*E. coli* O157:H7												
*L. monocytogenes*												
*Campylobacter*												
*C. cayetanensis*												

The second objective is to apply multi‐criteria decision analysis (MCDA) to evaluate and score each contamination source under defined risk criteria for each pathogen. These criteria could include (1) likelihood of contamination, (2) frequency of contamination, (3) environmental persistence (how long or how well do pathogens survive in a source before transfer), (4) pathway susceptibility (how many transport routes exist to transfer pathogens from a source to a produce farm), and (5) effectiveness of existing mitigation efforts. Overall, the output of Objective 2 is a ranked list of source criteria that can differentiate between sources that are frequently associated with contamination and those that occur less often but pose severe or persistent risks that are difficult to mitigate for each pathogen.

The third objective applies the RRM‐AL framework to real adjacent‐land scenarios, shifting the model from a conceptual risk‐ranking tool to a practical decision‐support system capable of evaluating contamination risks at the farm scale. This objective examines whether the source selection and the weighting and scoring structure in Objectives 1 and 2 perform as expected when tested against actual adjacent‐land settings, weather conditions, and management practices surrounding produce farms. The outputs from Objective 3 are risk profiles for each adjacent‐land scenario. These profiles can be used to identify which sources dominate a scenario, which sources escalate risk only under certain environmental conditions, and which sources require targeted mitigation or increased monitoring. They also provide a reproducible structure for anonymously comparing farms, documenting risk changes over time, or evaluating the impact of risk management strategies.

## 4 Conclusion

Adjacent and nearby land uses may be contributors to microbial contamination in produce production systems. Therefore, it is important for farms to perform an assessment of potential hazards adjacent to the farm as well as on‐farm. This review describes environmental dynamics, domesticated and wild animals, and human activities as key sources influencing potential contamination. Pathogens including *Salmonella*, *E. coli* O157:H7, *L. monocytogenes*, *Campylobacter*, and *C. cayetanensis* are shown to exploit multiple routes from runoff and irrigation water to dust and direct animal intrusion to move from adjacent lands onto produce farms. Despite these insights, existing studies often examine individual risk sources in isolation and lack an integrated framework to assess their combined impact under varying agricultural and ecological conditions. To bridge this gap, the proposed Risk‐Ranking Model for Adjacent Land (RRM‐AL) provides a structured, semi‐quantitative framework to evaluate and prioritize contamination sources from adjacent land. By integrating evidence from literature, surveillance, and expert elicitation through multi‐criteria decision analysis, the model ranks source‐pathogen pairs, evaluates contamination criteria and weighting distributions, and provides applications to real‐world produce farm scenarios.

## Author Contributions

**Tuan Le**: conceptualization, investigation, writing – original draft, writing – review and editing, methodology, formal analysis. **Joseph D. Eifert**: conceptualization, funding acquisition, supervision, resources, project administration, writing – review and editing. **Laura K. Strawn**: conceptualization, funding acquisition, writing – review and editing, project administration, supervision, resources.

## Conflicts of Interest

The authors declare no conflicts of interest.

## Data Availability

The data underlying this article will be shared at request to the corresponding author.
